# Degree of Hearing Improvement and Reduction of Air-Bone Gap After Tympanoplasty in a Tertiary Hospital in Saudi Arabia

**DOI:** 10.7759/cureus.55159

**Published:** 2024-02-28

**Authors:** Mohammed Al Hamoud, Atheer Alzubaidi, Khalid Al Shahrani, Ghaida Alotaibi, Faisal A Alkenani, Yahia Alahmari, Talat E Ardi, Mohammad Al-Ahmari, Mohammed Asiri

**Affiliations:** 1 Otolaryngology - Head and Neck Surgery, Aseer Central Hospital, Abha, SAU; 2 Otolaryngology - Head and Neck Surgery, Maternity and Children Hospital, Makkah, SAU; 3 Otolaryngology - Head and Neck Surgery, Maternity and Children Hospital, Jeddah, SAU; 4 Otolaryngology - Head and Neck Surgery, King Khalid University Hospital, Abha, SAU

**Keywords:** chronic suppurative otitis media, tympanic membrane perforation, ossicular chain, ossicles, tympanoplasty, csom: chronic suppurative otitis media, abg: air-bone gap

## Abstract

Background: A clinical condition known as chronic otitis media (COM) is characterized by tympanic membrane perforation, varying degrees of hearing loss, and otorrhea that lasts for two to six weeks. COM alone or with cholesteatoma may result in ossicular chain discontinuity and ossicular erosion. The hearing restoration procedure includes repairing the eardrum and building the ossicular chain in ears with damaged ossicles. Multiple studies suggest the predictive value of preoperative air-bone gap (ABG) to detect the ossicular chain status can help with proper preoperative planning for surgery.

Objective: To determine the degree of hearing improvement and reduction in ABG after tympanoplasty and to investigate the correlation between preoperative ABG and the status of the ossicular chain during surgery.

Study design, setting, and date: This retrospective hospital file-based study was conducted at Aseer Central Hospital, Southern Region, Saudi Arabia, between November 2022 and April 2023. Hospital records of patients who underwent tympanoplasty during 2018-2023 were reviewed. Eighty-five patients were diagnosed with chronic suppurative otitis media (CSOM) between 2018 and 2023. A data collection sheet was employed to record extracted data, including the patient’s age, sex, hearing assessment, type of surgical intervention, and outcome. We calculated the average of ABG decibels (dB) by summing the ABG values at 500 Hz, 1000 Hz, and 2000 Hz frequencies and dividing by three.

Results: In the present study, data from 85 patients who underwent tympanoplasty were analyzed. Approximately one-third of the patients were in the age group of 31 to 40 years (25, 29.4%), and 50 (58.8%) of them were females. Chronic medical conditions were observed in 30 (35.3%) patients, with diabetes being reported in 19 (63.3%) of those cases. CSOM was found to be present in the left ear of 47 (56.0%) patients. Among the patients, 25 (29.4%) had subtotal perforations, 12 (14.1%) had marginal perforations, and two (2.4%) had total tympanic membrane perforations. The majority of patients (67, 78.8%) exhibited conductive hearing loss, while the remaining 18 (21.2%) had mixed hearing loss. Of the patients, 13 (15.3%) and 20 (23.5%) had fixed and disrupted ossicular chains, respectively. In terms of ossicular disruption, incudostapedial joint (ISJ) fixing (21.2%), fixed stapes (18.2%), and ISJ dislocation (18.2%) were the most prevalent kinds. Prior to operations, the mean ± SD of ABG was 22.6 ± 7.5. ABG values were 19.0 ± 9.3 on average after surgery. The statistical difference between pre- and postoperative ABG was statistically significant (paired t-test, p = 0.007), with a mean difference of -3.7. There were no significant differences between the different statuses of ossicular chains and the type of tympanic membrane perforation.

Conclusion: This study suggests that the degree of preoperative ABG (dB) is a valuable predictor of intraoperative ossicular chain status and can aid in preoperative planning for ossicular chain reconstruction. Furthermore, the study found that the type of tympanic membrane perforation preoperatively is not a reliable indicator of the ossicular chain status. Finally, tympanoplasty is considered a beneficial surgical procedure with a significant improvement in hearing status postoperatively.

## Introduction

Chronic otitis media (COM) is a clinical condition marked by perforation of the tympanic membrane, varying degrees of hearing loss, and otorrhea lasting for at least two to six weeks [1]. Worldwide, it is estimated that between 65 million to 330 million people are affected by COM, with approximately 60% of them experiencing clinically significant hearing loss [1]. As the most common cause of adult conductive hearing impairment due to middle ear and eardrum damage, COM can occur with or without cholesteatoma, which is characterized by ossicular disruption caused by persistent inflammation in the tympanic cavity. Ossicular erosion, a common side effect of COM, could result in the complete breakdown of middle ear mechanics, leading to significant hearing impairment [2]. Repairing the eardrum and reconstructing the continuity of the ossicular chain in middle ears with damaged ossicles is a part of the hearing restoration procedure known as tympanoplasty [2].

Tympanoplasty, a surgical procedure aimed at repairing perforations in the tympanic membrane, is a commonly performed operation in otology surgical practice [3]. The primary objectives of this procedure are to prevent recurrent middle ear infections, improve hearing, and minimize the need for postoperative care [4,5]. The most common indications for tympanoplasty are hearing improvement and protection of the middle ear mucosa from infections via the external auditory canal [3]. Once the suppurative condition has been resolved in the middle ear cleft, and a dry ear has been achieved, tympanoplasty is performed to reconstruct the ossicular chain and repair the tympanic membrane perforation [6,7].

The purpose of tympanoplasty is twofold: to enhance the patient's hearing and to safeguard the middle ear against recurrent infections. Several studies have demonstrated significant improvements in hearing status following tympanoplasty. For instance, a study conducted by Lokhna et al. reported significant enhancements in hearing and the closure of the air-bone gap (ABG) after tympanoplasty [4].

The hearing thresholds can be influenced by various factors, including the size and location of the tympanic membrane perforation, ossicular chain erosion or discontinuity, as well as the presence of cholesteatoma and its growth patterns [8,9].

Although only intraoperative testing can determine whether ossicles are intact or eroded [2], in patients with COM, the preoperative results of the pure-tone average (PTA) and ABG decibel (dB) have been proposed as a viable approach to predict the status of the ossicular chain [1]. In a previous study conducted by Carrillo et al. [5], it was shown that the ABG in patients with COM is linked to the condition of the ossicular chain. Smaller values of the ABG predict ossicular integrity, while greater values (particularly at high frequencies) indicate ossicular discontinuity. In another study by Bayat et al. [1], when comparing the "intact ossicular" and "disrupted ossicular" groups, no statistically significant variations were found in ABG or PTA. Therefore, they concluded that ABG and PTA values do not always accurately reflect the condition of the middle ear transmission system. As a result, the aim of the current study is to identify the degree of hearing improvement after tympanoplasty and the correlation between preoperative ABG and ossicular chain status to assist surgeons in proper preoperative planning.

## Materials and methods

Objective

To determine the degree of hearing improvement and reduction in ABG (dB) after tympanoplasty and to investigate the correlation between preoperative ABG (dB) and the status of the ossicular chain during surgery.

Study design, setting, and date

This retrospective hospital file-based study was conducted at Aseer Central Hospital, Southern Region, Saudi Arabia, between November 2022 and April 2023. Hospital records of patients who underwent tympanoplasty during 2018-2023 were reviewed. Eighty-five patients were diagnosed with chronic suppurative otitis media (CSOM) between 2018 and 2023. A data collection sheet was employed to record extracted data, including the patient’s age, sex, hearing assessment, type of surgical intervention, and outcome. We calculated the average of ABG (dB) by summing the ABG (dB) values at 500Hz, 1000 Hz, and 2000 Hz frequencies and dividing by three.

The inclusion and exclusion criteria are presented in Table 1.

**Table 1:** Inclusion and exclusion criteria

Parameter	Category
Inclusion criteria	Patients who underwent tympanoplasty between January 2018 and November 2022 due to chronic ear disease
	All adults aged 18 years or more, male and females, from all races and ethnicities
	Patients with both preoperative and postoperative pure-tone audiometry
Exclusion criteria	Patients who underwent tympanoplasty before January 2018 and after November 2022
	Patients with incomplete clinical data
	Individuals aged less than 18 years old
	Individuals who underwent tympanoplasty outside the Aseer region
	Patients without preoperative pure-tone audiometry

Ethical considerations

The study protocol received approval from the Ethics Committee of Aseer Central Hospital, Aseer Institutional Review Board, Abha, Saudi Arabia (approval number: REC-02-01-2023). To ensure confidentiality, participant data were kept anonymous by assigning a unique code number to each patient on the data collection sheets.

Statistical analysis

Data analysis was conducted using RStudio (R version 4.3.0; Posit, Boston, MA). Categorical data were presented as frequencies and percentages, while numerical variables were represented as mean and standard deviation (SD). The reduction in ABG (dB) from preoperative to postoperative time points was assessed using a paired t-test. To examine statistical differences in ABG (dB) reduction between different patient groups, an independent samples t-test was utilized. Preoperative ABG (dB) differences across various types of tympanic membrane perforation were assessed using a one-way analysis of variance (ANOVA) test. The relationship between the site of tympanic membrane perforation and the status of the ossicular chain was evaluated using Fisher's exact test.

## Results

Demographic characteristics

In the current study, we analyzed data from 85 patients who underwent tympanoplasty. Almost one-third of the patients were aged 31 to 40 years (n = 25, 29.4%), and 58.8% (n = 50) of them were females. Chronic conditions were prevalent among 35.3% (n = 30) of the patients, of which diabetes was reported among 63.3% (n = 19) (Table 2).

**Table 2:** Demographic and preoperative characteristics of patients ^*^ Descriptive data are based on 30 patients who had chronic conditions. ^§^ The variable had one missing value. CSOM: chronic suppurative otitis media; CHL: conductive hearing loss.

Parameter	Category	N (%)
Age	<20	21 (24.7%)
	20 to 30	13 (15.3%)
	31 to 40	25 (29.4%)
	41 to 50	17 (20.0%)
	>50	9 (10.6%)
Gender	Male	35 (41.2%)
	Female	50 (58.8%)
Chronic conditions	Yes	30 (35.3%)
Type of chronic conditions^*^	Diabetes	19 (63.3%)
	Hypertension	7 (23.3%)
	Bronchial asthma	11 (36.7%)
Affected ear with CSOM^§^	Left	47 (56.0%)
	Right	37 (44.0%)
Site of tympanic membrane perforation	Central anterior	15 (17.6%)
	Central posterior	20 (23.5%)
	Central middle	11 (12.9%)
	Subtotal	25 (29.4%)
	Marginal	12 (14.1%)
	Total	2 (2.4%)
Presence of active ear discharge (infection) at the time of surgery	Yes	11 (12.9%)
Type of hearing loss in the affected ear	CHL	67 (78.8%)
	Mixed	18 (21.2%)
Degree of hearing loss	Mild	36 (42.4%)
	Moderate	33 (38.8%)
	Moderately severe	14 (16.5%)
	Severe	2 (2.4%)

Preoperative characteristics of the patients

CSOM was located in the left ear in 56.0% (n = 47) of patients. Subtotal, marginal, and total tympanic membrane perforations were evident among 29.4%, 14.1%, and 2.4% of the sample (n = 25, 12, and 2), respectively. The majority of patients (n = 67, 78.8%) had conductive hearing loss (CHL), and the remainder (n = 18, 21.2%) had mixed hearing loss. More details about the preoperative characteristics of the patients are listed in Table 2.

Operative characteristics

**Figure 1:**
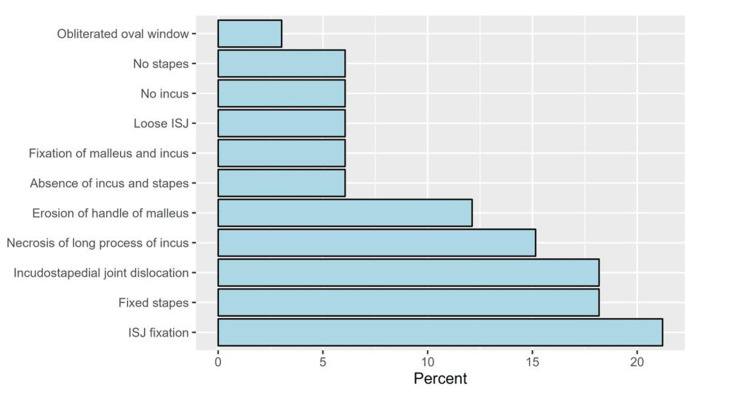
The percentage of types of ossicular chain disruption among patients with disrupted and fixed chains (n = 33) ISJ: incudostapedial joint.

Post auricular tympanoplasty was the most common surgical approach since it was performed for 70 patients (82.4%), whereas 17.6% (n = 15) of them underwent transcanal tympanoplasty. Temporalis fascia was the most frequent type of graft (n = 71, 83.5%). Fixed and disrupted ossicular chains were prevalent among 15.3% and 23.5% of patients (n = 13 and 20), respectively (Table 2). Among patients with fixed or disrupted ossicles (n = 33), the most common types of disruption were incudostapedial joint (ISJ) fixation (n = 7, 21.2%), fixed stapes (n = 6, 18.2%), and ISJ dislocation (n = 6, 18.2%, Figure 1). Ossiculoplasty was performed for 22.4% (n = 19) of the patients. Intraoperative characteristics are provided in Table 3.

**Table 3:** Intraoperative characteristics ^*^ Descriptive data are based on 19 patients who underwent ossiculoplasty. TORP: total ossicular replacement prosthesis: PORP: partial ossicular replacement prosthesis.

Parameter	Category	N (%)
Surgical approach used	Transcanal	15 (17.6%)
	Post auricular	70 (82.4%)
Type of graft used	Temporalis fascia	71 (83.5%)
	Cartilage	14 (16.5%)
Ossicular chain state	Intact	52 (61.2%)
	Fixed	13 (15.3%)
	Disrupted	20 (23.5%)
Ossiculoplasty	No	66 (77.6%)
	Yes	19 (22.4%)
Type of prosthesis used if ossiculoplasty done^*^	TORP	10 (52.6%)
	PORP	9 (47.4%)

Relationship between the site of tympanic membrane perforation and the status of the ossicular chain

There were no significant differences between different statuses of ossicular chains and having perforations in the central anterior, central posterior, and central middle regions. However, the proportion of patients with disrupted ossicular chains who had subtotal perforations was significantly lower than those with fixed (n = 6, 46.2%) and intact (n = 17, 32.7%) chains (p = 0.045). Furthermore, the proportion of patients with marginal perforations and intact chains (n = 0, 0.0%) was significantly lower than patients with fixed and disrupted chains (n = 4, 30.8% and n = 8, 40.0%, respectively; p < 0.001; Table 4).

**Table 4:** Relationship between the site of tympanic membrane perforation and the status of the ossicular chain

Site	Category	Ossicular chain status			p-value
		Intact, N = 52	Fixed, N = 13	Disrupted, N = 20	
Central anterior	No	39 (75.0%)	13 (100.0%)	18 (90.0%)	0.055
	Yes	13 (25.0%)	0 (0.0%)	2 (10.0%)	
Central posterior	No	40 (76.9%)	11 (84.6%)	14 (70.0%)	0.624
	Yes	12 (23.1%)	2 (15.4%)	6 (30.0%)	
Central middle	No	44 (84.6%)	12 (92.3%)	18 (90.0%)	0.814
	Yes	8 (15.4%)	1 (7.7%)	2 (10.0%)	
Subtotal	No	35 (67.3%)	7 (53.8%)	18 (90.0%)	0.045
	Yes	17 (32.7%)	6 (46.2%)	2 (10.0%)	
Marginal	No	52 (100.0%)	9 (69.2%)	12 (60.0%)	<0.001
	Yes	0 (0.0%)	4 (30.8%)	8 (40.0%)	
Total	No	50 (96.2%)	13 (100.0%)	20 (100.0%)	>0.999
	Yes	2 (3.8%)	0 (0.0%)	0 (0.0%)	

**Figure 2:**
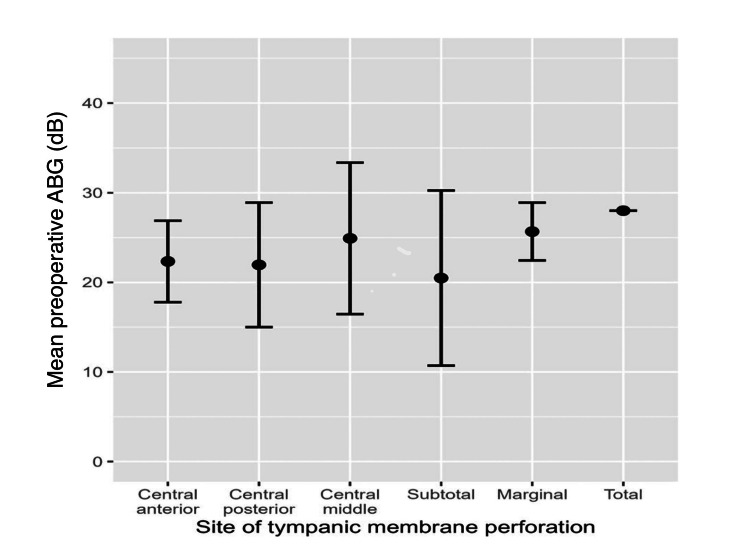
The mean preoperative ABG (dB) values across different sites of tympanic membrane perforation ABG: air-bone gap.

The degree of ABG (dB) before and after surgeries is as follows: the mean ± SD of ABG before surgeries was 22.6 ± 7.5. Preoperative ABG values differed significantly based on the ossicular chain status (20.1 ± 6.8 for intact, 23.8 ± 3.7 for fixed, and 28.6 ± 7.7 for disrupted, p < 0.001). Nevertheless, preoperative ABG did not differ significantly based on the types of tympanic membrane perforation (Figure 2).

**Figure 3:**
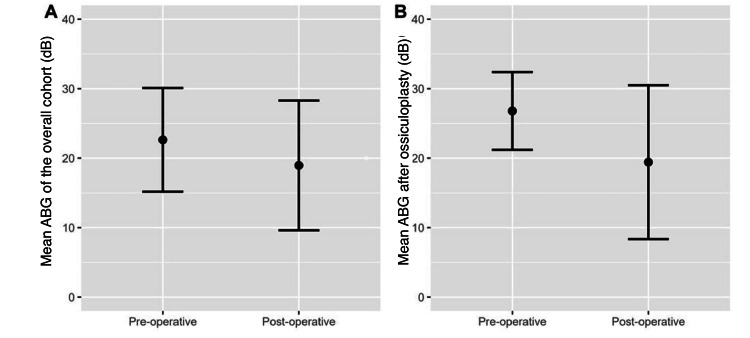
The mean ABG (dB) values before and after tympanoplasty Error bars represent standard deviations. ABG: air-bone gap.

The mean ± postoperative ABG value was 19.0 ± 9.3 (Figure 3A). The mean difference between pre and postoperative ABG was -3.7 (95% CI, -6.3 to -1.0). Normality testing of the ABG difference revealed a normally distributed variable (Shapiro-Wilk test, p = 0.189). The statistical difference was significant (paired t-test, p = 0.007). Focusing on patients who underwent ossiculoplasty, the mean (SD) ABG was reduced from the preoperative (26.8 ± 5.6) to the postoperative period (19.4 ± 11.1), and the mean change was statistically significant (mean change = -7.4, 95% CI, -13.3 to -1.5, paired t-test, p = 0.017, Figure 3B).

Concerning ABG reduction between groups, results showed no statistical differences between patients who underwent ossiculoplasty and those who did not (independent t-test, p = 0.147) and those who received temporalis fascia and cartilage grafts (independent t-test, p = 0.481) (Table 5).

**Table 5:** The difference in ABG (dB), reduction by ossiculoplasty, and the type of graft used ABG: air-bone gap.

Parameter	Category	ABG difference (dB)	p-value
Ossiculoplasty	No	-2.6 ± 12.2	0.147
	Yes	-7.4 ± 12.2	
Type of graft used	Temporalis fascia	-4.1 ± 12.4	0.481
	Cartilage	-1.6 ± 11.9	

## Discussion

Our study was conducted among patients diagnosed with CSOM who underwent tympanoplasty ± ossicular chain reconstruction. The primary aim of the study was to determine the extent of hearing improvement and the reduction in ABG following tympanoplasty. Additionally, we aimed to investigate the relationship between preoperative ABG and the condition of the surgical ossicular chain. In this retrospective study, we enrolled 85 patients who fully met the inclusion criteria. Approximately one-third of the patients were aged 31 to 40 years (n = 25, 29.4%), and 50 (58.8%) of them were females. The majority of patients (n = 67, 78.8%) had CHL, while the remaining (n = 18, 21.2%) had mixed hearing loss. This can be explained by the fact that one of the causes of CHL occurs when there is a disruption in the ossicular chain, tympanic membrane, or both, which aligns with the criteria of our study population.

By evaluating the middle ear exploration results, the frequency of osseous discontinuity and the association of ABG, and the degree of improvement and reduction of ABG following tympanoplasty, it will be possible to determine how frequently patients with CSOM experience ossicular chain erosion. A Qatar-based study [2] found that among the 279 ears, 66 (23.66%) of the ears had ossicular chain erosion, which was more common in the cholesteatoma ears (69.3%) than the safe CSOM ears (13.9%). The incus was the most frequently impaired ossicle (62 ears, 22.2%), while the malleus was the most resilient, eroded only in 13 (4.7%) ears, and the stapes was eroded in 31 (11.1%) ears. According to our findings, 38.8% of patients have ossicular abnormalities. Fixed and disrupted ossicular chains were prevalent among 15.3% and 23.5% of patients (n = 13 and 20), respectively. We found that among patients with fixed or disrupted ossicles (n = 33), the most common types of disruptions were ISJ fixation (n = 7, 21.2%), fixed stapes (n = 6, 18.2%), and ISJ dislocation (n = 6, 18.2%) [2,10].

Tympanic membrane perforations were visible in 29.4%, 14.1%, and 2.4% of the sample (n = 25, 12, and 2), respectively, subtotal, marginal, and total. No significant relationship was found between having different types of tympanic membrane perforation and preoperative ABG. This finding disagrees with the study done by Rana et al., which concludes the patient has greater ABG with an increased size of perforation with the greatest preoperative ABG found in patients with tympanic membrane perforation involving all four quadrants of the tympanic membrane (total perforation) [11].

There was a significant correlation between the ABG variable and hearing threshold in ossicular status scores in this study. A higher ABG and hearing threshold were associated with a higher ossicular status score, and vice versa. This difference was statistically significant (paired t-test, p = 0.007). Focusing on patients who underwent ossiculoplasty, the mean (SD) ABG decreased from the preoperative value (26.8 ± 5.6) to the postoperative period (19.4 ± 11.1), and this mean change was statistically significant (mean change = -7.4, 95% CI, -13.3 to -1.5, paired t-test, p = 0.017, Figure 3B).

Our findings are consistent with a study conducted in 2021 by Rizandiny et al., who found a strong correlation between hearing threshold levels and ossicular status scores. They concluded that there is a substantial and robust association between ABG scores and ossicular status scores. The ossicular status score increases in proportion to the ABG, and vice versa [12].

In our study, ABG was measured at 22.6 ± 7.5 prior to the operations. Depending on the state of the ossicular chain, the preoperative ABG levels varied significantly (20.1 ± 6.8 for intact, 23.8 ± 3.7 for fixed, and 28.6 ± 7.7 for disrupted, p = 0.001).

The postoperative ABG averaged 19.0 ± 9.3 (Figure 3A). The mean difference in ABG between the two time points was -3.7. These findings align with those of Lokhna et al., who reported a decrease in the ABG from a preoperative value of 46.62 ± 7.89 dB to a postoperative value of 23.43 ± 5.52 dB, demonstrating statistically significant improvements (p = 0.05) in postoperative pure tone audiometry [3].

Transcanal microscopic tympanoplasty with a cartilage graft, in contrast to postauricular tympanoplasty with a temporalis fascia graft, offers potential advantages such as reduced morbidity, less postoperative pain, improved patient comfort, a higher rate of graft acceptance, and shorter operative durations. In our study, postauricular tympanoplasty was the most commonly performed surgical technique, with 70 patients (82.4%) undergoing this procedure, while 17.6% (n = 15) of the patients opted for transcanal tympanoplasty.

In our study, we observed no significant difference between patients who received a temporalis fascia graft and those who received a cartilage graft. This finding aligns with the results of a study published in 2017 by Jain et al., who also reported no significant difference in postoperative ABG reduction between temporalis fascia grafts and cartilage grafts [13].

Following surgery, the average ABG was 19.0 ± 9.3 (Figure 3A). The average ABG difference between the two points was -3.7. These findings support those of Gokgoz et al., who demonstrated statistically significant improvements (p = 0.05) in ABG at all examined frequencies, as well as preoperative and postoperative pure tone audiometry [14].

One limitation of this study is the short duration of postoperative follow-up, which prevents us from knowing which of these findings will remain stable over time and which will change. Additionally, we did not compare which type of ossicular disruption was more associated with postoperative improvement and which was not. Therefore, well-conducted prospective studies with longer follow-up periods are necessary to confirm our results.

## Conclusions

In conclusion, this study suggests that the degree of preoperative ABG is a valuable predictor of intraoperative ossicular chain status and can aid in preoperative planning for ossicular chain reconstruction. Furthermore, the study found that the type of tympanic membrane perforation preoperatively is not a reliable indicator of the ossicular chain status. Finally, tympanoplasty is considered a beneficial surgical procedure with a significant improvement in hearing status postoperatively.
